# Prognostic role of tumour-associated macrophages and macrophage scavenger receptor 1 in prostate cancer: a systematic review and meta-analysis

**DOI:** 10.18632/oncotarget.18743

**Published:** 2017-06-27

**Authors:** Jian Cao, Jun Liu, Ran Xu, Xuan Zhu, Xiaokun Zhao, Bin-Zhi Qian

**Affiliations:** ^1^ Department of Urology, The Second Xiangya Hospital, Central South University, Changsha, Hunan 410011, P.R. China; ^2^ Department of Urology, The Fifth Affiliated Hospital of Xinjiang Medical University, Wulumuqi, Xinjiang 830011, P.R. China; ^3^ MRC Centre for Reproductive Health, Queen’s Medical Research Institute, Edinburgh EH16 4TJ, United Kingdom; ^4^ Current/Present address: Department of Urology, The Affiliated Cancer Hospital of Xiangya School of Medicine, Central South University, Changsha, Hunan 410013, P.R. China

**Keywords:** prostate cancer, tumor-associated macrophages, macrophage scavenger receptor 1, prognosis, meta-analysis

## Abstract

Recent studies suggested that the tumour associated macrophages may be associated with prostate cancer outcome. A meta-analysis was performed to evaluate the prognostic value of tumor associated macrophages and macrophage scavenger receptor 1, marker for a subset of macrophages, by pooled hazard ratio and 95% confidence intervals from qualified studies following a systemic search. The results indicate that higher infiltration of tumor associated macrophages predicts poor overall survival (HR=1.57, 95%CI: 1.15-1.98), but not biochemical recurrence (HR=1.01, 95%CI: 0.98-1.04) or recurrence-free survival (HR=1.03, 95%CI: 0.05-2.01). In contrast, elevated level of macrophage scavenger receptor 1 was significantly associated with better recurrence-free survival (HR=3.26, 95%CI: 1.22-5.29). Thus, our analysis confirmed the prognostic value of these markers in prostate cancer outcome. We also discussed potential causes of the controversies in the literature and future research directions.

## INTRODUCTION

Prostate cancer (Pca) is the second most common cancer and the fifth leading cause of cancer-associated death in men worldwide. It accounts for estimated 307,000 deaths, representing 6.6% of the total male cancer mortality in 2012 [1–3]. Although the use of prostate specific antigen (PSA), Gleason scores, cell cycle progression score and gene-expression profiles greatly advanced the diagnosis and prognosis [4–7], it is still challenging for urologists to make personalized clinical decisions due to the heterogeneity of the disease [8]. Therefore, more precise risk stratification and prognostic instruments are essential for more appropriate and effective clinical management and rational health resources distribution.

Macrophages, a type of innate immune cells, are a major component of tumour infiltrating immune cells. Traditionally, these cells are believed to be able to kill tumour cells based on *in vitro* experiments [9]. However, persuasive evidences from clinical and preclinical studies demonstrated that macrophages can promote cancer initiation, progression and metastasis. Tumor associated macrophages (TAMs) has been shown to influence tumour progression to different extend depending on tumour type [10]. TAMs infiltration has been shown to correlate with poor prognosis in cancers of breast, cervix, and bladder [11]. However, it correlates with better prognosis for non-small cell lung cancer [12, 13] and colorectal cancer [14, 15], which suggests distinct mechanisms in different tumour types and/or different tissue environment.

Many studies have been performed to look into macrophage associated markers in Pca samples with various cohort sizes and end points [16–20]. However, the results remain controversial. For example, Lissbrant et al reported that the high volume density of TAMs predicts worse cancer specific survival [21], whereas Shimura et al reported that total macrophage density was associated with recurrence free survival [17]. High density of CD68^+^ macrophages have been shown to predict an increased risk of biochemical recurrence in one study [22], but not in another study [20]. Thus, it is important to perform a meta-analysis and systemic review to assess these controversies.

Macrophage scavenger receptor (MSR1, also known as CD204, SR-A) is a marker of alternatively activated macrophages [23] and is involved in host immune responses [24]. Nonsense and missense mutations of MSR1 were identified to be associated with increased Pca risk [25]. Intriguingly, recent clinical studies indicate that increased MSR1 expression is correlated with good prognosis in Pca [26, 27]. In current study, we systematically reviewed the relevant literature and performed a meta-analysis to determine the prognostic value of the level of TAMs and MSR1 in Pca. We also discussed potential causes of controversies in these clinical studies and future research directions.

## RESULTS

### Selection and characteristics of included studies

**Figure 1:**
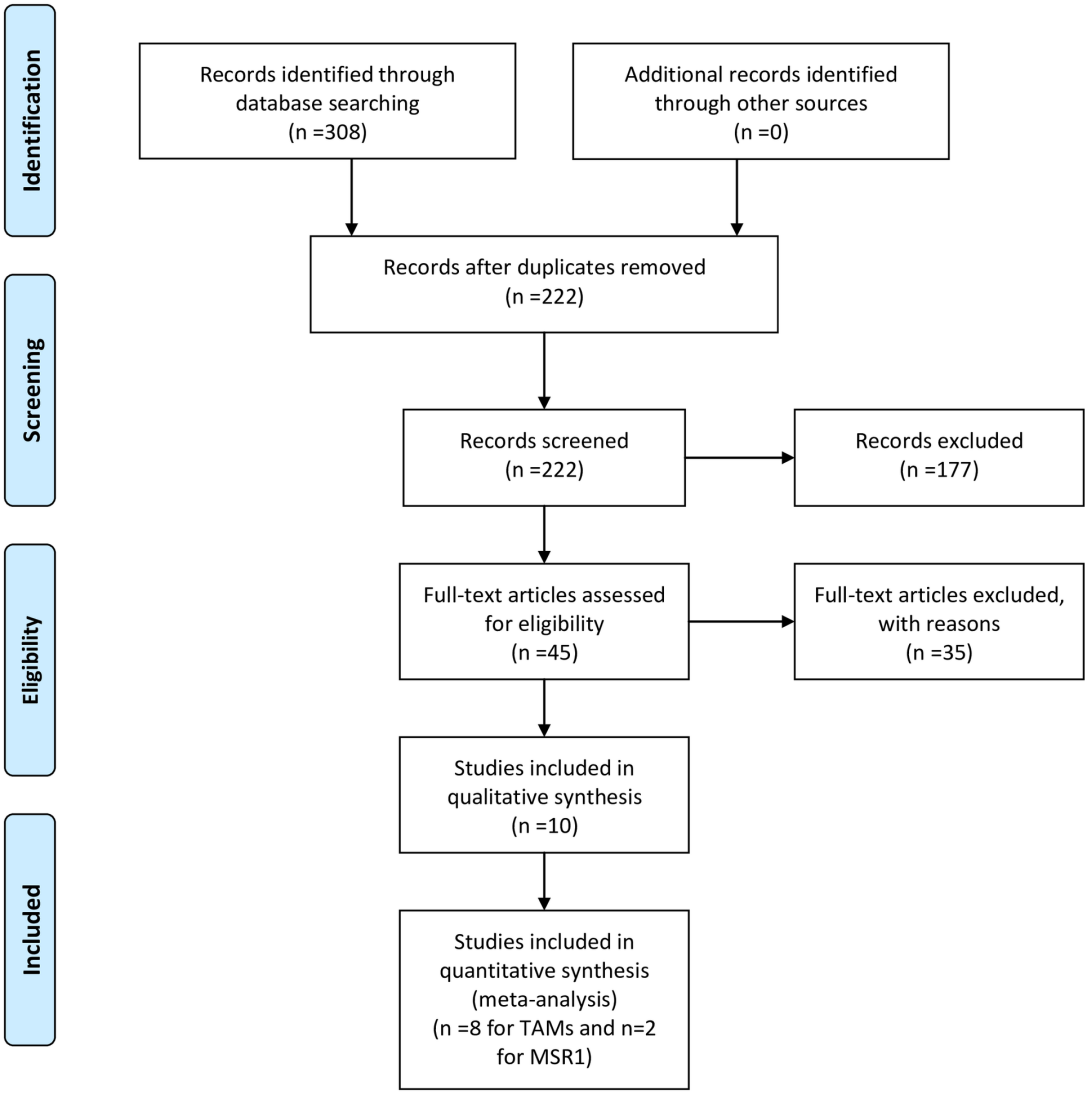
Flow diagram of literature search and selection for meta-analysis **(A)** A flow chart of the literature search and selection of included studies for TAMs. **(B)** A flow chart of the literature search and selection of included studies for MSR1.

The flow chart of the literature search is shown in Figure 1. The initial search algorithm retrieved a total of 173 studies related to TAMs and 135 studies related to MSR1 and the prognosis of Pca. Based on the inclusion/exclusion criteria (detailed in Materials and Methods), 8 studies on TAMs [16–22, 28] and 2 studies on MSR1 [26, 29] published between 2000 and 2016 were included in our meta-analysis. As the studies by Gollapudi et al and Gannon et al each included two independent cohorts with separate HR and 95%CI, they were marked as Gollapudi et al West LA, Gollapudi et al Durham VA, and Gannon et al CTR, Gannon et al ADT.

The characteristics of the included studies are summarized in Table 1 and Supplementary Table 1. In TAMs evaluation, a total of 1084 patients were included. TAMs were detected by the immunohistochemistry or immunofluorescence staining against CD68 as a marker. In MSR1 evaluation, 2 cohorts with a total of 213 patients were included. MSR1 was detected by immunohistochemistry staining two different monoclonal antibodies against the same protein.

**Table 1:** The characteristics of the included studies

Author	Year	Country	Number	Data type^1^	Cut-off^2^	Treatments^3^	HR (95%CI)
**TAMs and OS**							
Hu et al,	2015	China	42	Multivariate	Mean>29.43/HPF	RP	1.52(1.16-2.01)
Lissbrant et al,	2000	Sweden	85	Kaplan-Meier/Multivariate	Ave volume density ≥0.97%	TUR	2.5(1.24-5.02)
**TAMs and BCR**							
Gollapudi et al, *West LA*	2013	USA	332	Multivariate	Mean>6.6/core	RP	1.04(0.99-1.1)
Lanciotti et al,	2014	Italy	93	Multivariate	Mean>15.3/hot spot	RP	2.53(1.6-9.67)
Gannon et al, CTR	2009	Canada	40	Univariate	Mean>0.598/unit	RP	4.26(1.39-13.07)
Gannon et al, ADT	2009	Canada	35	Univariate	Mean>1.066/unit	RP/ADT	1.29(0.40-4.21)
Gollapudi K *Durham VA*	2013	USA	205	Multivariate	Mean>6.8/core	RP	1(0.97-1.03)
**TAMs and RFS**							
Alev et al,	2015	Turkey	100	Univariate	Mild/Moderate/strong	RP	0.69(0.18-2.80)
Shimura et al,	2000	USA	81	Kaplan-Meier/Multivariate	Mean>185.8/mm^2^	RP	0.46(0.21-0.99)
Nonomura et al,	2010	Japan	131	Multivariate	Mean>22/HPF	ADT	2.69(1.46-5.04)
Lanciotti et al,	2014	Italy	93	Kaplan-Meier/Univariate	Mean>15.3/hot spot	RP	1.86(0.44-7.94)
**MSR1 and RFS**							
Yang et al,	2004	USA	78	Multivariate	Mean>72.8/mm^2^	RP	4.93(1.95-12.42)
Takayama et al,	2008	Japan	135	Multivariate	Mean>24/HPF	RP/Rad	2.96(1.48-5.89)

1. The statistical methods used to get HRs and 95%CIs.

2. High density TAMs or MRS1 defined as the mean count of cells more than cut-off.

3. RP-radical prostatectomy; TUR-transurethral resection; ADT-androgen depletion treatment; Rad-radiotherapy.

### Data synthesis

#### TAMs and overall survival

**Figure 2:**
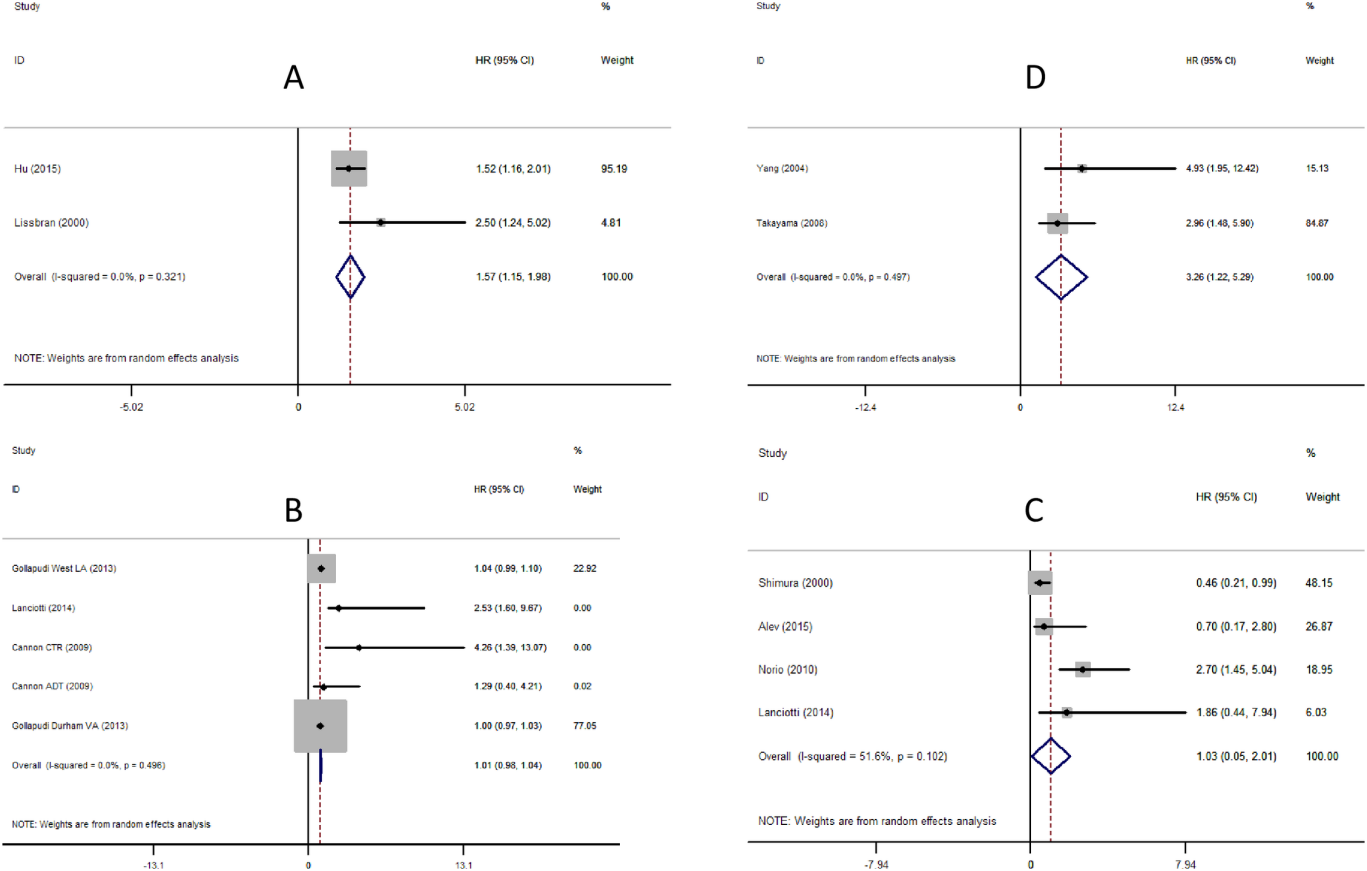
Meta-analysis of the association between TAMs, MSR1 and prognosis of Pca Each study was shown by the name of the first author (publish year) and the HRs with 95%CIs. **(A)** Forrest plot and meta-analysis of studies evaluating HR of high density of TAMs and overall survival. **(B)** Forrest plot and meta-analysis of studies evaluating HR of high density of TAMs and biochemical recurrence. **(C)** Forrest plot and meta-analysis of studies evaluating HR of high density of TAMs and recurrence-free survival. **(D)** Forrest plot and meta-analysis of studies evaluating HR of high density of MSR1 and recurrence-free survival.

The pooled estimates demonstrated a significant association between high density of TAMs and a worse prognosis regarding overall survival (Figure 2A). (HR=1.57, 95%CI: 1.15-1.98).

#### TAMs and biochemical recurrence

As shown in Figure 2B, TAM density showed no statistical significance in predicting biochemical recurrence (HR=1.01, 95%CI: 0.98-1.04). Sub-group analysis according to the sample number: A group (n >100, HR=1.01, 95%CI: 0.98-1.05) and B group (n <100, HR=1.74, 95%CI: 0.08-3.39); or according to therapy: Surgery group (HR=1.01, 95%CI: 0.98-1.04) and ADT group (HR=1.29, 95%CI: -0.61-3.19) revealed no statistical significance (Supplementary Figures 1 and 2 respectively).

#### TAMs and recurrence-free survival

No significant correlation was observed between TAMs density and recurrence-free survival (HR=1.03, 95%CI: 0.05-2.01) (Figure 2C).

#### MSR1 and recurrence-free survival

The incidence of recurrence-free survival was 3.26-fold higher in patients with high density of MSR1 than those with the low density (HR=3.26, 95%CI: 1.22-5.29) (Figure 2D).

#### Evaluation of publication bias

Both Begg’s test and Egger’s test were used and did not reveal any evidence of significant asymmetry in the present meta-analysis. Because of limited numbers of studies, we employed “trim and fill” analysis to evaluate the publication bias of TAMs and OS, MSR1 and RFS, which is more sensitive than Begg’s test and Egger’s test [30, 31]. In TAMs studies with OS as end point, the pooled HR did not change significantly when calculated assuming one missing study (1.52, 95%CI: 1.20-1.94) (Supplementary Figure 3). Similarly, assuming one missing study on MRS1 and RFS, the pooled HR is 2.96 (95%CI: 1.84-4.76) (Supplementary Figure 4). Both data indicate that the result of our analysis is stable.

## DISCUSSION

Our meta-analysis indicates that higher density of TAMs is associated with poorer overall survival, but not with biochemical recurrence or recurrence-free survival in Pca. Gollapudi et al indicated TAMs number in Pca was significantly higher than prostatic intraepithelial neoplasia and benign, and also higher in Gleason grade 4 compared with Gleason grade 3 [20]. Additionally, Hu et al reported that TAM infiltration in prostate tumour increased in patients with metastasis compared with those no metastasis [19]. Furthermore, using an optimized computer-assistance quantification approach, Gannon et al [22] reported that TAM number increased significantly in Pca patients received androgen deprivation treatment (ADT) compared with patients without ADT. Together, these studies suggest that the density of TAMs increased in prostate cancer tissues along Pca progression which suggest a tumour promoting role of TAMs in Pca.

Several preclinical studies suggested potential mechanisms of TAMs promotion in Pca progression. Fang et al indicated that macrophages can induce the prostate tumorigenesis through activation of the AR-CCL4-STAT3 axis signaling [32]. Comito et al demonstrated the cross talks among cancer-associated fibroblasts (CAFs), TAMs and Pca cells. Prostate CAFs induce monocytes recruitment and TAMs differentiation through stromal-derived growth factor-1, and TAMs in turn induce activation of fibroblasts. Together, CAFs and M2 macrophages promote tumour cell motility and metastasis, as well as de novo angiogenesis through activation of endothelial cells [33]. In Chen et al’s study, Pca-secreted CCN3 has been shown to recruit macrophages and skew their differentiation to a CD206+ M2 phenotype which in turn contribute to VEGF-dependent angiogenesis [34]. Similarly, Kwon et al reported that TAMs could be induced by Pca-derived BMP-6 to produce IL-1a, which, in turn, promotes angiogenesis and Pca growth [35]. Moreover, Lee et al indicated that BMP-6 increased macrophage interleukin-6 expression and promoted Pca castration resistance [36].

Therefore, targeting macrophages may have important applications for Pca treatment. CC chemokine 2 (CCL2)/CC chemokine receptor 2 (CCR2) and Colony Stimulating Factor 1 (CSF1) receptor signaling are major pathways that regulate macrophage recruitment and function *in vivo*. Preclinical studies showed that increased CCL2 expression in Pca cells induces macrophage recruitment to protect Pca cells from docetaxel-induced cytotoxicity and enhance metastasis [37, 38]. Targeting CCL2 with neutralizing antibodies inhibits Pca growth and bone metastasis [38, 39]. CSF1 expression in Pca cells can be induced by ADT and radiotherapy which led a significant increase in TAMs infiltration [40, 41]. Small molecule inhibitors of CSF1 receptor enhanced the efficacy of ADT and radiotherapy in Pca preclinical models [40, 41]. Reagents that target CCL2/CCR2 and CSF1R pathways are currently being tested in clinical trials (clinicaltrials.gov).

It should be noted that TAM infiltration are usually not homogeneous within the tumor. Different areas of tumour mass were chosen to quantify macrophages density in the included studies. Among the eight included studies, five studies used randomly selected fields within cancer cores to calculate the mean number of macrophages, whereas hot spots were used in two studies. Shimura et al reported the prognosis data using the density of macrophages in cancer core, stroma, cancer, and hot spot. Densities of macrophages in these four areas were used individually with other pathological markers in multivariate analyses; while only the density of macrophages in cancer cores is significantly associated recurrence-free survival. Together, these data suggest that TAMs infiltration in the tumour core may have tumour promoting function which invites further investigation in mechanistic studies.

MSR1 is considered to be a marker for alternatively activated macrophages, or M2. Many studies indicated that these macrophages often promote tumour progression and therapy resistance. In inflammation associated cancers, pro-inflammatory macrophages, or M1, have been shown to promote tumour initiation. Interestingly, in present study, we found that the density of MSR1 expression was inversely correlated with better recurrence free survival in Pca. A higher level of MSR1 density was associated with lower clinical stage, positive lymph nodes, smaller tumour size, and lower preoperative PSA level [26] and good prognosis of Pca [29]. MSR1 has a broad range of functions due to a wide range of ligands that they can bind together with various co-receptors [24]. For example, elevated expression of MSR1 and its co-receptor MERTK have been shown to enhance macrophage function in clearance of apoptotic cells and suppression of inflammation [42]. On the other hand, MSR1 expression level can be suppressed by inflammatory cytokines such as transforming growth factor beta1 and/or interleukin 6 [43, 44]. Our data suggest that MSR1 labels a subset of anti-tumour TAMs in Pca, which may inhibit tumour progression. It should be noted that M1/M2 is a rather oversimplified way of describing macrophage polarization. TAMs often exhibit mixed phenotypes and undergo phenotypic switch upon stimulation. Thus, it will be interesting to further explore TAM polarization and heterogeneity by using additional markers that are associated with TAM functions in order to further understand the disease mechanism and design personalized treatment.

There are some limitations in this meta-analysis. Firstly, several studies without enough survival data for data aggregation were excluded. Secondly, only studies using anti-CD68 antibodies to detect the TAMs were included. Studies detecting subpopulations of TAMs using markers such as CD163 or CD169 were not included in this meta-analysis. Thirdly, variation in staining protocol, histological analysis and scoring criteria among different studies were inevitable. Larger-scale multi-center prospective studies under standard experimental design, measurement method and uniform definition are needed to minimize the heterogeneity and reach a prognostic standard.

In summary, our studies indicate that the density of TAMs and MSR1 are promising markers for prediction of clinical outcome of Pca. This can provide important implications for clinical management and rational health resources distribution. MSR1 may serve as an important marker in identifying TAMs subset to help understand disease mechanism and improve prognosis. More functional markers should be employed to better define different TAM subpopulations in order to further investigate their roles in Pca.

## MATERIALS AND METHODS

### Search strategy

This meta-analysis follows the standard protocol proposed by Preferred Reporting Items for Systematic Review and Meta-Analysis [45]. A systematic search was performed in PubMed, Embase, and Cochrane Library for all studies using terms related to Pca (e.g. prostate neoplasms, or prostate carcinoma, or prostate tumour, or prostatic cancer) and terms related to tumour-associated macrophages (e.g. tumour infiltrating macrophages, or intratumoural macrophages) or macrophage scavenger receptor (e.g. acetyl-LDL receptors, or scavenger receptor). Both Medical subheadings (MeSH) and free text words were applied as keywords. A search filter from McMaster University of Health Information Research Unit was applied in retrieved results from PubMed or Embase with the best balance of sensitivity and specificity for prognostic studies (http://hiru.mcmaster.ca/hiru/HIRU_Hedges_MEDLINE_Strategies.aspx). In order to minimize publication bias, we also searched China National Knowledge Infrastructure (CNKI), a Chinese academic database. No relevant study was found.

The last search was updated on 16^st^ February 2017 and bibliographies of the relevant articles were explored to prevent missing studies by electronic search strategies. The search was conducted by two authors independently, and any discrepancies were resolved through iteration and consensus.

### Inclusion and exclusion criteria

All candidate articles were scanned by two independent investigators (Jian and Jun). Divergences were solved by group discussion. The inclusion criteria were: (a) confirmed diagnosis of Pca with or without metastasis; (b) immunohistochemistry or immunofluorescence staining were used to detect TAMs and MSR1; (c) correlation between TAMs, MSR1 and survival data in Pca were reported; (d) HR and 95% CI were provided or can be reconstructed from data provided. Exclusion criteria were as follows: (a) reviews, case reports, abstracts, letters or editorials; (b) studies without sufficient sample size to reconstruct HR and 95% CI; (c) endpoints used in only one study; (d) articles written in language other than Chinese or English.

### Data extraction

Data was extracted independently by two investigators (Jian and Jun), and any disagreement was resolved by group debate. The following information was collected from each study: first author’s name, country of the study population, patient numbers, year of publication, age of the population studied, study design, sampling time span, cut-off value of the density of TAMs or MSR1, follow up time, markers used for TAMs or MSR1, type of statistical analysis, primary endpoint, and HRs with 95%CI of different endpoints reported in at least two studies. Biochemical recurrence was considered as PSA >0.2 ng/mL on two consecutive measurements after the first radical medical treatments. Quality assessment of each studies was independently performed according to the Dutch Cochrane Centre proposed by Meta-analysis of Observational Studies in Epidemiology (MOOSE) by two investigators.

### Statistical analysis

Hazard ratio and 95% confidence intervals were obtained directly from each study or from reconstruction according to methods described by Tierney et al [46]. If univariate and multivariate analyses were available, multivariate analysis data was chosen for the meta-analysis, which was considered superior to univariate analysis. Due to prior assumptions of the heterogeneity between primary studies, we also performed meta-analysis using the random-effects model that is more conservative. Publication bias of studies was analyzed by the Begg’s funnel plot and the Egger’s linear regression test and p value <0.05 was considered significant. The “trim and fill” methods [31] was used to evaluate the influence of publication bias on the overall effect. All statistical analysis were conducted with the STATA software version 12.0 (STATA Corporation, College Station, TX, USA). All statistical tests were two-sided and the significance level was set at 5%.

## SUPPLEMENTARY MATERIALS FIGURES AND TABLE




