# Supplementary material for: Prognostic role of tumour-associated macrophages and macrophage scavenger receptor 1 in prostate cancer: a systematic review and meta-analysis
Source: Oncotarget. 2017 Jun 27;8(47):83261–9. doi: 10.18632/oncotarget.18743 (PMC5669966; doi:10.18632/oncotarget.18743)
Supplement: Supplementary file 1 [file oncotarget-08-83261-s001.pdf]

Prognostic role of tumour-associated macrophages and macrophage scavenger receptor 1 in prostate cancer: a systematic review and meta-analysis

SUPPLEMENTARY MATERIALS

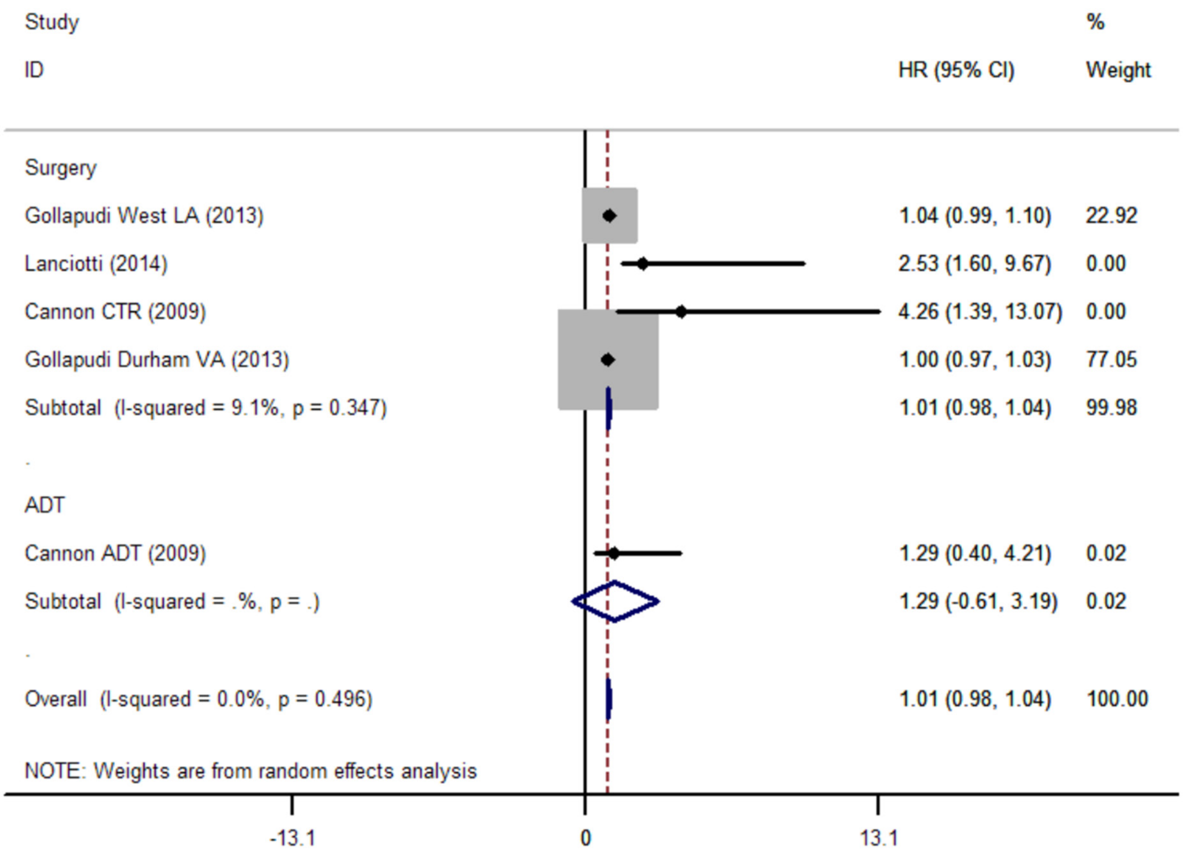

Supplementary Figure 1: Forrest plot and subgroup meta-analysis of TAMs and BCR: Surgery VS Androgen depletion treatment (ADT).

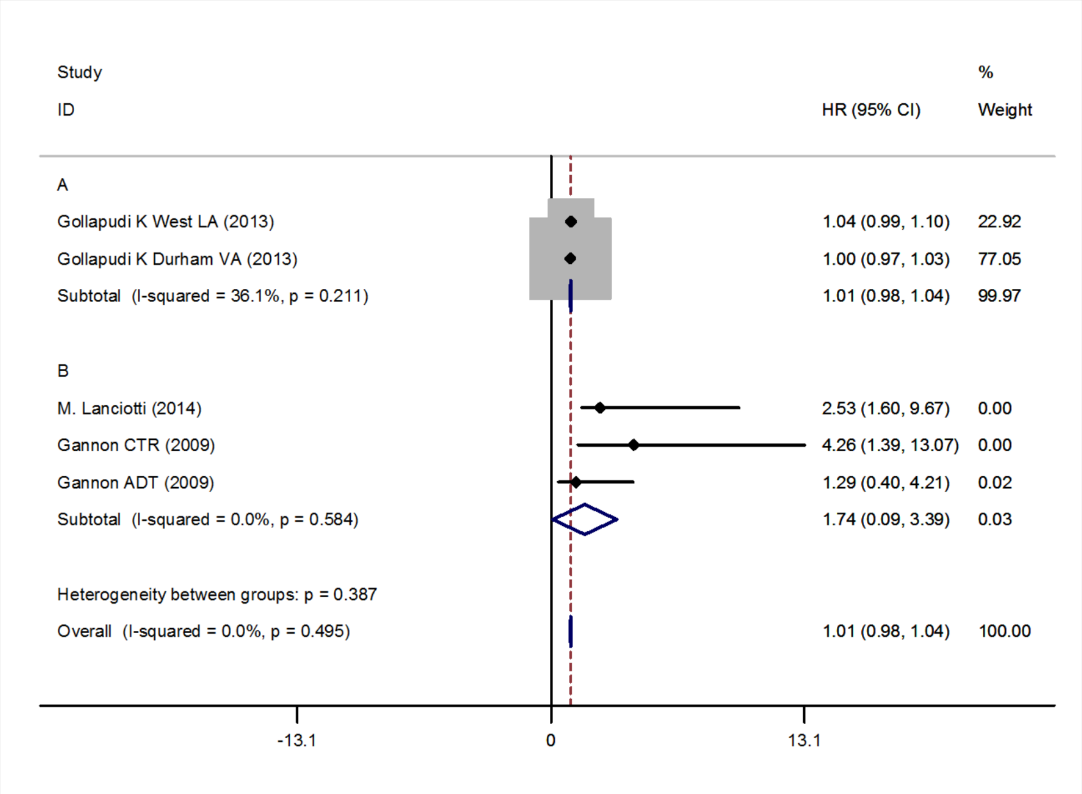

Supplementary Figure 2: Forrest plot and subgroup meta-analysis of TAMs and BCR: A (n >100) VS B (n <100).

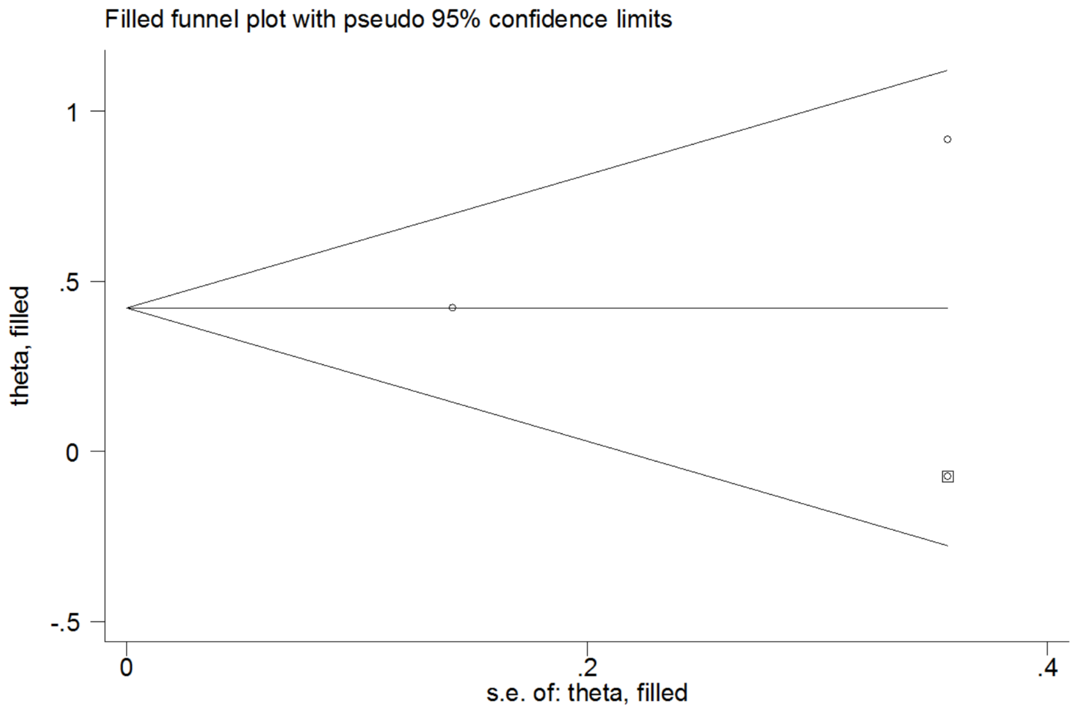

Supplementary Figure 3: "trim and fill" analysis of the source of publication bias for TAMs and OS.

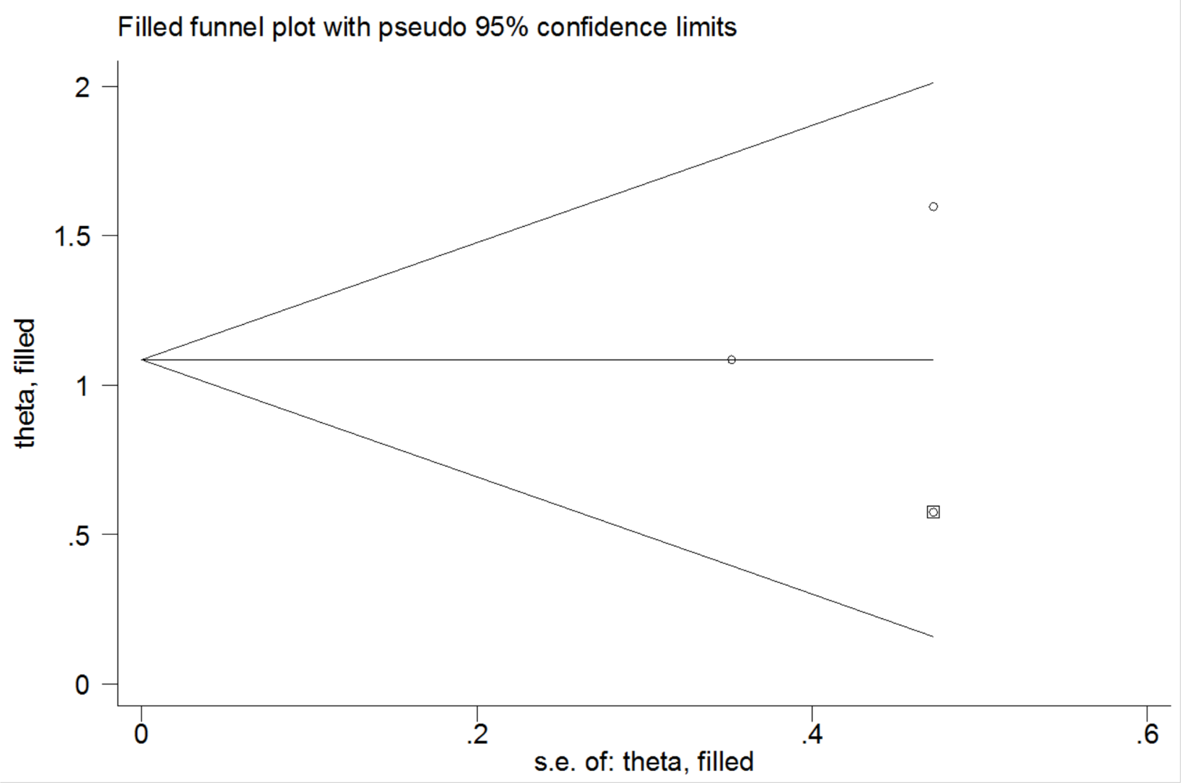

Supplementary Figure 4: "trim and fill" analysis of the source of publication bias for MSR1and RFS.

Supplementary Table 1: Characteristics of included studies

See Supplementary File 1
